# Brain glutamate concentration in men with early psychosis: a magnetic resonance spectroscopy case–control study at 7 T

**DOI:** 10.1038/s41398-021-01477-6

**Published:** 2021-06-17

**Authors:** Beata R. Godlewska, Amedeo Minichino, Uzay Emir, Ilinca Angelescu, Belinda Lennox, Masa Micunovic, Oliver Howes, Philip J. Cowen

**Affiliations:** 1 Department of Psychiatry, University of Oxford, Oxford, UK; 2 Oxford Health NHS Foundation Trust, Oxford, UK; 3 Early Intervention Psychosis NHS England South, Oxford, UK; 4 Wellcome Centre for Integrative Neuroimaging, University of Oxford, Oxford, UK; 5 Department of Psychosis Studies, Institute of Psychiatry, Psychology and Neuroscience, London, UK

**Keywords:** Schizophrenia, Neuroscience

## Abstract

Abnormalities in glutamate neurotransmission are linked to psychotic symptoms and cognitive dysfunction in schizophrenia. magnetic resonance spectroscopy (MRS) provides an acceptable means of measuring glutamate in the human brain but findings from patient studies at conventional magnetic field strength show considerable heterogeneity. Ultra-high-field MRS offers greater precision in glutamate measurement, particularly in delineation of glutamate from its precursor and metabolite, glutamine. This study aimed to use high-field (7 T) MRS to measure concentrations of glutamate and glutamine in three brain regions, anterior cingulate cortex (ACC), dorsolateral prefrontal cortex (DLPFC) and putamen (PUT), in young men with early psychosis. MRS was performed in 17 male participants with early psychosis and 18 healthy age-matched controls. Neurometabolite levels were calculated with unsuppressed water signal as the reference and corrected for individual grey matter, white matter and cerebrospinal fluid concentration. Cognitive function was measured with the Brief Assessment of Cognition in Schizophrenia (BACS). Compared to controls, patients with early psychosis had lower concentrations of glutamate and glutamine in ACC. No differences were apparent in the DLPFC and PUT. In patients with early psychosis, there was a highly significant correlation between glutamate concentration in ACC and performance on the BACS, though the numbers available for this analysis were small. Our finding of lower glutamate levels in ACC in patients with schizophrenia is consistent with a recent meta-analysis of 7 T studies and suggests that this abnormality is present in both patients with early psychosis and those with longer-established illness. The possible link between ACC glutamate and cognitive performance requires replication in larger studies.

## Introduction

The role of glutamate neurons in the pathophysiology of schizophrenia is attracting intense research interest [1,2,]. Magnetic resonance spectroscopy (MRS) is a non-invasive means of quantifying glutamate concentration in the living human brain, and numerous MRS studies have measured glutamate levels in patients with schizophrenia. Levels of glutamine, the precursor and metabolite of glutamate have also been reported in some investigations.

A meta-analysis of 59 case–control studies (1686 patients and 1451 healthy controls) found no difference between patients with schizophrenia and controls in glutamate and glutamine levels in frontal brain regions [3]. However, in patients with schizophrenia, glutamate levels were increased in basal ganglia while levels of glutamine were elevated in thalamus. These findings suggest that the impact of schizophrenia on brain glutamate activity may show distinct regional specificity. It has also been suggested that abnormalities in glutamate might alter during the course of the illness with elevated levels being apparent at onset of psychosis, while lower levels supervene in patients with more established illness [4].

Most of the extant studies have been carried out at 3 T [3]. MRS at 7 T has the ability to provide a clearer delineation of glutamate from glutamine spectra than MRS at 3 T and offers the possibility of providing more reliable measures of glutamate activity in psychiatric studies [5]. A recent meta-analysis assessed nine case–control studies conducted at 7 T in patients with schizophrenia (255 patients and 293 healthy controls) [6]. The majority of neurometabolite measurements were made in anterior cingulate cortex (ACC). About half the patients were experiencing a first episode of illness and the great majority was treated with antipsychotic medication. The authors concluded that there were significant but modest reductions in glutamate but not glutamine in ACC in patients with schizophrenia. Interestingly, a reduction in brain glutathione (GSH) was also apparent. GSH is the chief cellular antioxidant in the brain and low GSH levels have been linked to glutamate excitotoxicity in schizophrenia [4]. However, only one 7 T study examined glutamate in striatal areas where 3 T studies have suggested that glutamate levels might be increased [3,7,].

The aim of the present study was to use MRS at 7 T to assess levels of glutamate, glutamine and GSH in patients with early psychosis and age and gender-matched controls, in three brain regions, pregenual ACC, dorsolateral prefrontal cortex (DLPFC) and putamen (PUT), that have been linked to psychotic symptoms and cognitive dysfunction in schizophrenia [8–10]. We predicted that patients with early psychosis would demonstrate a decrease in glutamate and GSH in frontal brain regions and an increase in glutamate in the PUT. We also assessed the cognitive function of participants using the Brief Assessment of Cognition in Schizophrenia (BACS, [11]) to explore potential relationships between MRS glutamate levels and cognitive performance.

## Patients and methods

### Participants and clinical ratings

The study was approved by the National Research Ethics Committee South Central—Oxford C. Participants with psychosis were recruited through Early Intervention in Psychosis service, Oxford Health NHS Foundation Trust, and Department of Psychosis Studies, Institute of Psychiatry, London, from patients diagnosed with early psychosis by their treating psychiatrist, independent of the research team, according to the Diagnostic and Statistical Manual for Mental Disorders Fifth Edition (DSM-5) criteria [12]. Healthy participants were recruited through the Department of Psychiatry volunteer register and word of mouth. Overall, 17 patients with early psychosis and 18 healthy controls, all male, were included in the study after giving full informed written consent.

The inclusion criteria for patients were: first episode psychotic illness as defined by Melbourne criteria (i.e. a week or more of psychotic symptoms above a threshold of moderate in severity), mental state stable enough to allow study participation and for both participant groups, being able and willing to give informed consent. The exclusion criteria were substance dependence as defined by DSM-5, clinically significant risk of suicidal behaviour, contraindication to MRS imaging and claustrophobia. Healthy controls were required to be free from any current or past history of significant psychiatric disorder on DSM-5 and not taking any psychotropic medications.

Symptom presence and severity were measured by the Positive and Negative Syndrome Scale (PANSS), allowing an assessment of positive, negative and general psychopathology in psychosis [13]. Cognitive function was tested with the BACS [11,14,]. The BACS measures global cognitive function across six domains: verbal memory with word list learning task, working memory with digit sequencing task, verbal fluency with controlled oral word association task, motor function with token motor task, attention and speed of information processing with symbol coding task and executive functions with tower of London task (see [14] for more detailed description of the tasks). Together they comprise a composite score. Scores are corrected for age and gender using stratified norms and expressed as a *z*-score [14].

### MRS

**Fig. 1:**
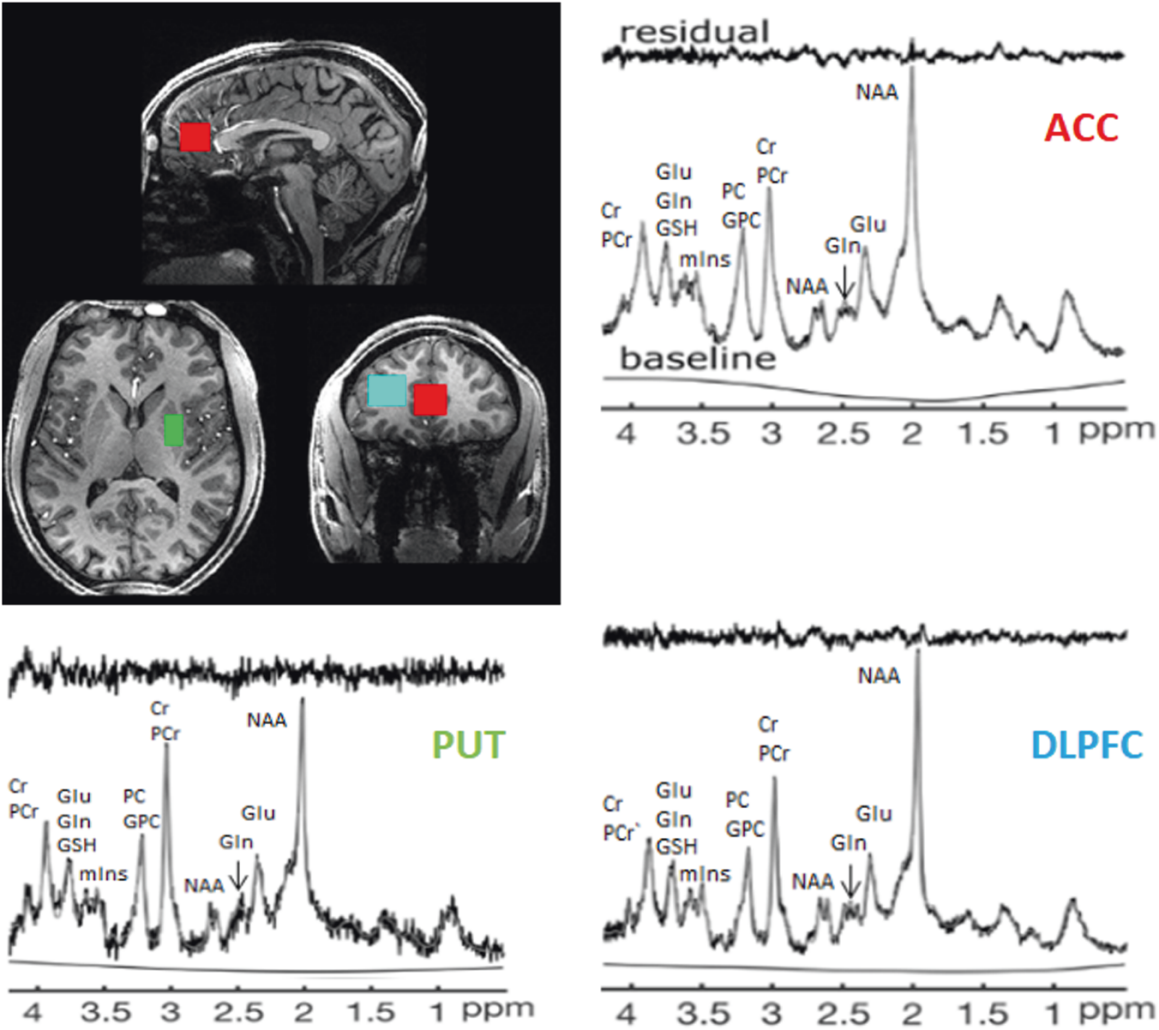
Voxel placement and representative spectra from the ACC (red), PUT (green) and DLPFC (DLPFC). Glu glutamate, Gln glutamine, GSH glutathione, Cr creatine, PCr phosphocreatine, myoIns myo-inositol, PC phosphocholine, GPC glycerophosphocholine, NAA N-acetylaspartate, Asc ascorbate.

^1^H-MRS scanning was used for the study, with all participants being scanned at the Functional Magnetic Resonance Imaging of the Brain Centre in Oxford. A 7-T Siemens MAGNETOM scanner with a Nova Medical 32 channel receive array head coil was employed in order to measure neurometabolites of three voxels of interest, in the ACC (20 × 20 × 20 mm), DLPFC (15 × 15 × 30 mm) and PUT (10 × 16 × 20 mm) (Fig. 1). This order of spectra acquisition was maintained for all but one patient, in whom the ACC measurement was repeated at the end of the scan due to shimming difficulties during the initial acquisition. These voxels were manually placed by referencing to a high-resolution 1 mm T1-weighted MP RAGE image. Firstly, gradient-echo shimming was used to adjust first- and second-order shims [15], followed by fine adjustments of first order shims with FASTMAP [16]. Spectra were obtained through a stimulated echo acquisition mode pulse sequence (TE = 11 ms, TR = 5 s, number of transients = 64 for the ACC and PUT = 32 for the DLPFC) with variable power radiofrequency pulses with optimised relaxation delays (VAPOR) water suppression and outer volume saturation [17] (see Fig. 1 for example spectra). Correction of residual eddy current effects and reconstruction of the phased array spectra was conducted with unsuppressed water spectra from the same, above mentioned, voxels.

### MRS processing

Neurometabolites were quantified with LCModel [18], with prior reported chemical shifts and coupling constants [19,20,] as a basis for the model spectra of glutamine, glutamate, and GSH by using GAMMA/PyGAMMA simulation library of VESPA for applying the density matrix formalism (Versatile Simulation, Pulses and Analysis 9). The same RF pulses and sequence timing, like those on the 7-T system, were used to perform simulations. Further, the model spectra included a macromolecule spectrum acquired from the occipital cortex, using an inversion recovery sequence (TR = 3 s, TE = 11 ms, inversion time TI = 0.685 s). The concentration of the neurometabolites was acquired relative to an unsuppressed water spectrum obtained from the same VOI [21].

The MP RAGE images were segmented using SPM to determine grey matter (GM), white matter (WM) and cerebrospinal fluid (CSF) fraction (fGM, fWM, fCSF) in the voxels [22]. The results of segmentations were visually inspected. Concentrations were then corrected for these with the following formula:

$$\begin{array}{l}{\mathrm{MetCorr}}\\ = ({\mathrm{MetConcAbs}} \times ({\mathrm{fGM}} \times 43300 + {\mathrm{fWM}} \times 35880 + {\mathrm{fCSF}} \times 55556)) /\left( {1 - {\mathrm{fCFS}}} \right)\end{array}$$

where MetCorr is the corrected concentration of a metabolite, MetConcAbs is this metabolite concentration from the LCModel output and fGM, fWM and fCSF stand for, respectively, fractions of GM, WM and CSF in the voxel of interest.

In order to allow comparability with other relevant studies (i.e. [23,24,]), metabolites quantified with the Cramér–Rao lower bounds (CRLB, estimated error of the metabolite quantification) ⩾30% were classified as not detected.

### Statistical analysis

Statistical analyses were performed with SPSS (version 23, IBM Corp, Armonk, NY). Differences between the patient group and the healthy control group were determined using independent samples *t*-tests or *χ*^2^ tests. The planned comparisons in neurometabolites between the two participant groups were glutamate, glutamine and GSH. Correlations were made using Pearson’s product moment. No correction was made for multiple comparisons.

## Results

At the time of the scan, all but two patients were receiving treatment with antipsychotic medication, which included olanzapine, risperidone, aripiprazole, quetiapine, clozapine, zuclopenthixol, lurasidone and paliperidone (see Supplementary Table 1 for details). Six patients were additionally treated with antidepressants (sertraline, mirtazapine and venlafaxine) and one with pregabalin. The patient and healthy control groups did not differ significantly in terms of age (Table 1). In the early psychosis group, the mean duration of psychosis onset was 30.5 months (range 11–55). As expected, the PANSS scores of the patients were significantly higher than the controls while their performance on the BACS was uniformly lower. Nine BACS datasets were available for patients and fifteen BACS datasets for healthy volunteers. Eight patients recruited from London missed BACS due to restrictions related to ethical approvals and three healthy volunteers chose to only have an MRS scan performed. One of the patients for whom BACS scores were available did not have the ACC MRS data, which left eight patients who had both BACS data and ACC MRS data.

**Table 1:** Demographic data and clinical scores^a^.

	Patients with early psychosis, *n* = 17	Healthy controls, *n* = 18	*t* value	*p* value
Age	25.6 (1.1)	27.1 (0.8)	−1.10	0.281
Time since illness onset (months)	30.5 (3.4)	–	–	–
Smoking	4/17	0/18	4.782 (*χ*^2^)	0.029
Cannabis use within 3 months of testing	3/17	1/18	1.263 (*χ*^2^)	0.261
Antipsychotic drugs	15/17	–		
Antidepressant drugs (in all cases concomitant to antipsychotics)	7/17	–		
PANSS				
Total	58 (2.5)	30 (0.0)	11.50	0.000
Positive	14.5 (0.8)	7 (0)	9.73	0.000
Negative	14.4 (1.0)	7 (0)	7.29	0.000
General	29.1 (1.2)	16 (0)	11.17	0.000
BACS				
Composite *z*-score	−0.61 (0.47)	0.99 (0.21)	−3.57	0.002
Verbal memory (word list learning)	−0.14 (0.37)	0.93 (0.21)	−2.68	0.014
Working memory (digital sequencing)	−0.90 (0.37)	0.083 (0.20)	−2.58	0.017
Verbal fluency (oral word association)	−0.06 (0.44)	1.10 (0.34)	−2.09	0.049
Attention and speed of information processing (symbol coding)	−0.62 (0.55)	0.53 (0.23)	−3.45	0.002
Executive function (tower of London)	−0.29 (0.40)	0.61 (0.17)	−2.40	0.025
Motor function (token motor task)	89.33 (12.17)	118.00 (8.3)	−2.01	0.057

^a^Values are mean (SEM).

MRS data were available for all but one patient who was unable to complete the scan due to high levels of anxiety. One scan was rejected for reasons of quality based on full width at half maximum (FWHM) = 0.133 (see Supplementary Table 2 for details of FWHM). Due to patient related and technical issues, including excessive motion and difficulties shimming, we were unable to obtain ACC and DLPFC measurements from one patient and ACC data from another. DLPFC and PUT measurements were not obtained from one patient each. In three healthy controls PFC data were not obtained. All remaining spectra were of good quality and there were no between-group difference in scan quality measures (*p* > 0.05). For results describing the signal-to-noise ratio and FWHM, see Supplementary Information.

GM, WM and CSF content did not differ between the early psychosis group and controls in any of the voxels (all > 0.05, see Supplementary Table 3 for details). Moreover, there was no significant difference between the groups in CRLB values for any of the metabolites tested (see Supplementary Table 4 for details). For all reported metabolites, CRLB values were <20% single measurements and no data were excluded on the basis of this criterion.

**Fig. 2:**
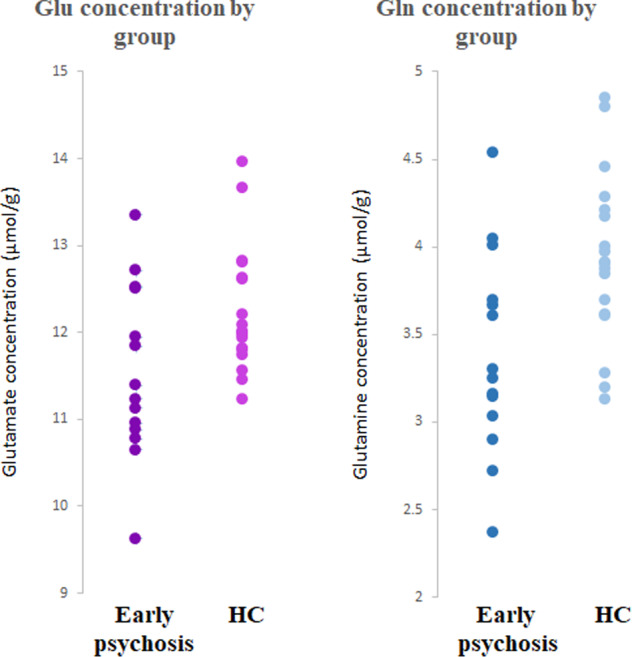
Glutamate (Glu) and glutamine (Gln) concentrations in the ACC by group. In the ACC, concentrations (in μmol/lg) of both glutamate (Glu) and glutamine (Gln) were significantly lower in patients with early psychosis (n=14) than healthy controls (HC, n=18) (respectively, p=0.029 and 0.007).

In the ACC, concentrations of both glutamate and glutamine were significantly lower in patients with early psychosis than controls (Table 2, Fig. 2). In DLPFC and PUT, there were no significant differences in concentrations of glutamate and glutamine between the two participant groups, though a trend to lower glutamine was seen in DFPLC in early psychosis (Table 2). There were no differences in GSH concentrations between patients with early psychosis and controls in any of the three voxels.

**Table 2:** Mean (SEM) absolute concentrations (μmol/g) of glutamate, glutamine and GSH, corrected for GM, WM and CSF content in ACC, DLPFC and PUT.

Region	Metabolite	Patients with early psychosis, *n* ACC = 14, *n* PUT = 16, *n* DLPFC = 14	Healthy controls, *n* ACC = 18, *n* PUT = 18, *n* DLPFC = 16	*t* value	*p* value	Cohen’s *d* effect size
ACC	Glutamate	11.54 (0.27)	12.24 (0.17)	2.30	0.029	0.80
	Glutamine	3.39 (0.16)	3.93 (0.11)	2.90	0.007	1.01
	GSH	1.38 (0.09)	1.29 (0.06)	−0.90	0.377	0.31
DLPFC	Glutamate	8.87 (0.17)	9.00 (0.65)	0.57	0.571	0.21
	Glutamine	2.48 (0.14)	2.80 (0.10)	1.92	0.065	0.69
	GSH	1.30 (0.07)	1.30 (0.06)	−0.05	0.963	0.02
PUT	Glutamate	9.57 (0.24)	9.67 (0.24)	−0.27	0.793	0.09
	Glutamine	3.22 (0.19)	3.01 (0.15)	0.78	0.440	0.01
	GSH	1.25 (0.09)	1.43 (0.72)	1.61	0.117	0.59

*n* number of datasets included in the final analysis.

**Fig. 3:**
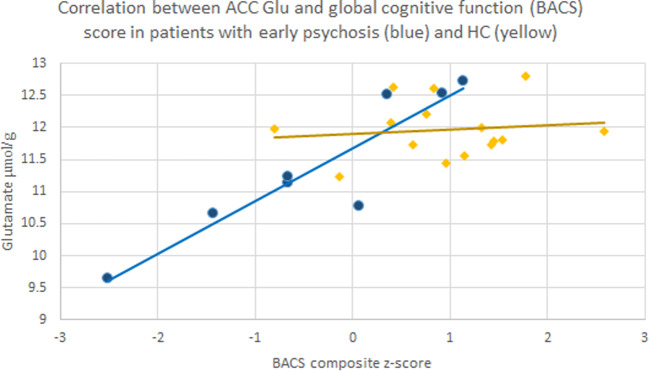
Correlation between ACC glutamate concentration and BACS score. Correlation between individual glutamate (Glu) concentrations (μmol/g) in the ACC of patients with first episode psychosis (blue), healthy controls (HC,yellow) and BACS composite z-score, representing global cognitive function; early psychosis r = 0.92, p = 0.001; HC r = 0.13, p = 0.64.

In the patients with early psychosis, there was no significant correlation in any of the voxels between glutamate, glutamine and GSH concentrations and total PANSS score, as well as PANSS subscales scores, evaluating positive, negative and general symptoms (all *p* > 0.05). Significant negative correlations were seen between the duration of the psychotic illness and the BACS score (−0.86; *p* = 0.003, *n* = 9), concentration of GSH in the PUT (−0.74; *p* = 0.002, *n* = 14) and concentration of glutamate in DLPFC (−0.60, *p* = 0.029, *n* = 13). There was also a highly significant correlation between glutamate levels in ACC and composite BACS score (0.92, *p* = 0.001, *n* = 8) (Fig. 3). This correlation was not significant in the healthy controls (*r* = 0.13, *p* = 0.64, *n* = 15).

Significantly more patients than healthy controls smoked cigarettes (Table 1). Given the reports on the effect of smoking on MRS glutamate measures [25], we performed additional univariate ANOVAs on glutamate and glutamine concentrations, where ‘smoking’ was introduced as an additional between subjects factor to ‘diagnosis’ (patients with early psychosis vs. healthy controls). Including smoking status did not have a significant main effect on glutamate and glutamine levels in any of the three regions tested (*p* > 0.05), while a main effect of diagnosis was maintained for both glutamate and glutamine in the ACC (respectively, *F* = 8.56, *p* = 0.007; *F* = 7.44, *p* = 0.011), though the reduction in glutamate was now more highly significant.

## Discussion

The main finding of this 7 T MRS study was that, compared to healthy controls, patients with early psychosis had lower concentrations of glutamate and glutamine in the ACC. There were no differences, however, in levels of glutamate and glutamine between patients and healthy controls in DLPFC and PUT. In addition, in patients with early psychosis, concentrations of glutamate in the ACC correlated with cognitive function as measured by the BACS, although the numbers available for this analysis were very small. Finally, there were no differences in GSH concentrations between patients and controls in any of the voxels studied.

Our findings are consistent with the meta-analysis of 7 T MRS studies in schizophrenia reported by Sydnor and Roalf [6] in finding lower glutamate levels in ACC relative to healthy participants. Nevertheless, there have been negative studies of this measure at 7 T [26,27,]. It has been suggested that levels of glutamate may change during the course of psychotic illness and perhaps be related to symptomatic status [4,28,]. Clearly longitudinal, within-subject studies would be the best way to assess this. However, ACC glutamate levels were lower in our early psychosis patients and a similar abnormality was reported by Kumar et al. [4] in patients with established ‘residual schizophrenia’ who exhibited prominent negative symptoms and functional impairment, in the absence of significant positive symptoms. Whether elevated glutamate levels in ACC might be seen in 7 T studies at patients at high risk of psychosis or patients with treatment resistance [29,30,] are important questions for future study.

The definition of first episode psychosis (FEP) varies between studies [31] and our patients had a longer duration of illness (mean 30.5 months) than those in other 7 T MRS studies of FEP patients where the average illness duration was around 12 months [23,24,32,]. For this reason, for our patient group we have used the term early psychosis rather than FEP. The fact that these studies also found lowered glutamate in ACC suggests that once psychosis is established, the duration of illness is not a critical determinant of glutamate levels in this brain region.

### Interpretation of findings

MRS studies at 7 T have an advantage in their ability to resolve the spectral peaks of glutamate and glutamine with greater precision than at lower field strengths [5]. In our study, the lowering in glutamine level in ACC in early psychosis was more prominent than the reduction in glutamate (about 14% versus 6%) and a similar finding was seen in the study of Kumar et al. [4] in patients with longer-established schizophrenia. However, in the 7 T meta-analysis [6], overall there was no lowering of glutamine, so this finding is likely to be less consistent than the reduction in glutamate and perhaps more related to individual study characteristics.

Interpretation of glutamate changes in MRS studies is challenging because MRS cannot distinguish between neuronal glutamate linked to neurotransmission and that involved in cellular metabolic processes such as the synthesis of GSH and proteins [33]. Similar comments with regard to cellular metabolic processes apply to glutamine, though, since much glutamine is derived from the uptake of synaptically released glutamate, our findings are consistent with decreased synaptic glutamate activity in first episode and early psychosis patients. The likely cause of this is not clear but hypotheses have linked low glutamate in schizophrenia to a process of excitotoxicity, which leads ultimately to the loss of glutamatergic synapses [4,28,34,]. Clearly, if this is the explanation for lowered glutamate in the ACC of patients with early psychosis, such a process must begin relatively early in the disease. Excitotoxicity has been linked to depletion of GSH [4] and, while this was not apparent in our study, overall the 7 T studies suggest that cortical GSH is lower in patients with schizophrenia [6].

Impaired cognition is an important feature of schizophrenia which is present in the ‘high-risk’ state and so can precede the development of overt psychosis [35]. The glutamatergic system plays a key role in several domains of cognition [36] but how far MRS glutamate in schizophrenia correlates with cognitive deficits is not clear [37]. In this context, our finding in participants with early psychosis of a significant correlation between performance on the BACS and glutamate levels in ACC is intriguing, albeit in a small number of participants with early psychosis and so requires replication. It is noteworthy that in large sample of FEP patients (*n* = 81), Wang et al. [24] in a 7 T study found only a modest positive correlation between verbal memory and glutamate level in ACC and no correlation between this glutamate measure and a composite score of cognitive performance. Interestingly, we did not observe a similar relationship between glutamate and cognition in healthy controls. It might be argued that in healthy people, who do not have low glutamate levels, glutamatergic activity is not a limiting factor in cognitive performance.

Findings from 3 T studies indicate that in schizophrenia glutamate and glutamine levels might be increased in subcortical brain areas [3]. This question has been little studied at 7 T but our findings agree with those of Thakkar et al. [7] who found no change in glutamate in the striatum of patients with established schizophrenia and Wang et al. [24] who found no increase in either glutamate or glutamine in the thalamus. It may be, however, that increases in glutamate in schizophrenia are more apparent in those patients with a high level of treatment resistance [38].

### Limitations and strengths

The modest sample size is an important limitation to our study. Although sample sizes similar to ours are not uncommon in 7 T MRS FEP and early psychosis research (e.g. [23,32,]), another recent study included 81 patients with FEP [24]. Larger samples increase power and the statistical reliability of findings [39]. Another significant limitation of our study is the medication status of patients. All but two of our patients were taking antipsychotic medication and there was also quite frequent use of antidepressant medication. While in a study in depressed patients we found no effect of SSRI treatment on MRS measures of glutamate and GSH [40], in a meta-analysis of 37 studies, Kubota et al. [41] reported that antipsychotic drug treatment was associated with a significant reduction in frontal glutamate concentrations compared to the pre-treatment state. Consistent with this, a meta-analysis of 13 MRS studies conducted by Kaminski et al. [42] found that while overall there was no difference in glutamate measures in DLPFC between patients with schizophrenia and healthy controls, increased levels of glutamate could be discerned in those patients who were antipsychotic naïve. These findings increase the importance of further MRS studies in unmedicated patients.

Another limitation is that our study investigated male participants only. While this may have increased sample homogeneity, it means that we cannot necessarily extrapolate our findings to women with early psychosis. In previous 7 T MRS studies of FEP, about one quarter of the patients were female and sex was not a significant moderating factor in the meta-analysis reported by Sydnor and Roalf [6]. It is important to note that the DLPFC voxel contained a relatively low proportion of GM which is reflected in the DLPFC segmentation. Even though we corrected for this in our calculation of the neurometabolites concentrations, the findings from this brain region should be received with caution.

The strengths of the current study include high-field MRS and inclusion of multiple regions, among them the PUT, a striatal region which has been linked to increased glutamate levels in 3 T studies in patients with schizophrenia. In addition, both patients and controls were carefully ascertained and assessed with reliable instruments.

## Conclusions

Our observations with glutamate are consistent with the composite findings of 7 T MRS studies in schizophrenia and suggest that glutamate levels are lowered in ACC in both patients with early psychosis and those with longer-established illness. Some patient groups also demonstrate lower levels of glutamine and GSH and further work is needed to identify the clinical and study characteristics that correlate with these abnormalities. It is intriguing that impaired cognitive performance in patients may correlate with lowered glutamate concentration in ACC. This could allow more specific targeting of glutamatergic treatments to alleviate the cognitive dysfunctions associated with schizophrenia [43].

## Supplementary information

Supplementary table 1

Supplementary table 2

Supplementary table 3

Supplementary table 4
