# Supplementary material for: Brain glutamate concentration in men with early psychosis: a magnetic resonance spectroscopy case–control study at 7 T
Source: Transl Psychiatry. 2021 Jun 17;11:367. doi: 10.1038/s41398-021-01477-6 (PMC8257573; doi:10.1038/s41398-021-01477-6)
Supplement: Supplementary file 1 — Supplementary table 1 [file 41398_2021_1477_MOESM1_ESM.docx]

Supplementary Table 1. Medications and their daily doses taken by patients with early psychosis.

| Participant | Antipsychotics at the time of testing | Daily dose | Other medications | Dose |
| --- | --- | --- | --- | --- |
| 1 | Olanzapine | 10mg |  |  |
| 2 | Clozapine | 250mg | Venlafaxine MR | 150mg |
| 3 | Lurasidone | 37mg | Sertraline | 50mg |
|  |  |  | Mirtazapine | 45mg |
| 4 | Quetiapine | 300mg |  |  |
| 5 | Quetiapine | 300mg | Sertraline | 100mg |
| 6 | risperidone | 4mg | Sertraline | 100mg |
| 7 | Olanzapine | 12.5mg | Sertraline  Mirtazapine  Pregabalin | 200mg  45mg  600mg |
| 8 | Olanzapine | 10mg |  |  |
| 9 | Zuclopenthizol  Aripiprazole | 6mg  10mg |  |  |
| 10 | Riperidone | 3mg |  |  |
| 11 | Aripiprazol | 10mg |  |  |
| 12 | Risperidon | 3mg |  |  |
| 13 | Aripiprazole | 15mg |  |  |
| 14 | Paliperidone | 100mg/monthly |  |  |
| 15 | Olanzapine | 15mg | Sertraline | 50mg |
| 16 | Drug-free |  |  |  |
| 17 | Drug-free |  |  |  |
