# Supplementary material for: Brain glutamate concentration in men with early psychosis: a magnetic resonance spectroscopy case–control study at 7 T
Source: Transl Psychiatry. 2021 Jun 17;11:367. doi: 10.1038/s41398-021-01477-6 (PMC8257573; doi:10.1038/s41398-021-01477-6)
Supplement: Supplementary file 2 — Supplementary table 2 [file 41398_2021_1477_MOESM2_ESM.docx]

Supplementary Table 2. Mean full-width half maximum (FWHM) (SEM) and signal-to-noise ratio (SNR) (SEM) of ACC, DLPFC and PUT; n - number of datasets included in the final analysis.

|  | Patients with early psychosis  n ACC = 14  n PUT=16  n DLPFC=14 | Healthy controls  n ACC = 18  n PUT=18  n DLPFC=16 | t value | p value |
| --- | --- | --- | --- | --- |
| FWHM_ACC | 0.037 (0.002) | 0.037 (0.001) | 0.290 | 0.774 |
| FWHM_DLPFC | 0.031 (0.001) | 0.033 (0.002) | -0.871 | 0.391 |
| FWHM_PUT | 0.044 (0.002) | 0.045 (0.002) | -0.402 | 0.690 |
| SNR_ACC | 35.93 (1.48) | 34.50 (1.11) | 0.789 | 0.437 |
| SNR_DLPFC | 33.57 (1.60) | 35.00 (1.61) | -0.626 | 0.537 |
| SNR_PUT | 14.81 (0.55) | 14.67 (0.69) | 0.871 | 0.894 |
