# Supplementary material for: Brain glutamate concentration in men with early psychosis: a magnetic resonance spectroscopy case–control study at 7 T
Source: Transl Psychiatry. 2021 Jun 17;11:367. doi: 10.1038/s41398-021-01477-6 (PMC8257573; doi:10.1038/s41398-021-01477-6)
Supplement: Supplementary file 3 — Supplementary table 3 [file 41398_2021_1477_MOESM3_ESM.docx]

Supplementary Table 3. Grey matter (GM), white matter (WM) and cerebro-spinal fluid (CFS) content in in ACC, DLPFC and PUT (SEM); n - number of datasets included in the final analysis.

|  | Patients with early psychosis  n ACC = 14  n PUT=16  n DLPFC=14 | Healthy controls  n ACC = 18  n PUT=18  n DLPFC=16 | t value | p value |
| --- | --- | --- | --- | --- |
| GM_ACC | 0.808 (0.016) | 0.834 (0.013) | -1.263 | 0.216 |
| GM_DLPFC | 0.334 (0.023) | 0.377 (0.017) | -1.555 | 0.131 |
| GM_PUT | 0.370 (0.025) | 0.345 (0.018) | 0.808 | 0.425 |
| WM_ACC | 0.108 (0.009) | 0.093 (0.003) | 1.719 | 0.096 |
| WM_DLPFC | 0.656 (0.023) | 0.607 (0.015) | 1.834 | 0.077 |
| WM_PUT | 0.636 (0.024) | 0.654 (0.018) | -0.604 | 0.550 |
| CFS_ACC | 0.082 (0.013) | 0.072 (0.013) | 0.538 | 0.594 |
| CFS_DLPFC | 0.009 (0.002) | 0.008 (0.002) | 0.099 | 0.992 |
| CFS_PUT | 0.000 (0.000) | 0.000 (0.000) | 1.063 | 0.296 |
