# Supplementary material for: Brain glutamate concentration in men with early psychosis: a magnetic resonance spectroscopy case–control study at 7 T
Source: Transl Psychiatry. 2021 Jun 17;11:367. doi: 10.1038/s41398-021-01477-6 (PMC8257573; doi:10.1038/s41398-021-01477-6)
Supplement: Supplementary file 4 — Supplementary table 4 [file 41398_2021_1477_MOESM4_ESM.docx]

Supplementary Table 4. Mean (SEM) Cramer-Rao Lower Bound (CRLB) of glutamate and glutamine in ACC, DLPFC and PUT; n - number of datasets included in the final analysis.

| Region | Metabolite CRLB | Patients with early psychosis  n ACC = 14  n PUT=16  n DLPFC=14 | Healthy controls  n ACC = 18  n PUT=18  n DLPFC=16 | t value | p value |
| --- | --- | --- | --- | --- | --- |
| ACC | Glutamate | 2.07 (0.071) | 2.06 (0.056) | 0.178 | 0.860 |
|  | Glutamine | 7.14 (0.553) | 6.28 (0.394) | 1.308 | 0.201 |
|  | GSH | 9.36 (0.836) | 9.50 (0.326) | -0.174 | 0.863 |
| DLPFC | Glutamate | 2.79 (0.579) | 2.38 (0.619) | 1.868 | 0.72 |
|  | Glutamine | 10.21 (2.259) | 8.63 (1.996) | 2.046 | 0.05 |
|  | GSH | 10.64 (1.243) | 9.75 (0.661) | 0.657 | 0.517 |
| PUT | Glutamate | 3.75 (0.577) | 3.89 (0.676) | -0.640 | 0.527 |
|  | Glutamine | 13.31 (3.911) | 13.33 (4.524) | -0.014 | 0.989 |
|  | GSH | 17.06 (1.662) | 14.44 (0.837) | 1.455 | 0.155 |
